# Correction of Vertebral Bone Development in Ectodysplasin A1-Deficient Mice by Prenatal Treatment With a Replacement Protein

**DOI:** 10.3389/fgene.2021.709736

**Published:** 2021-08-11

**Authors:** Clara-Sophie Kossel, Mandy Wahlbuhl, Sonia Schuepbach-Mallepell, Jung Park, Christine Kowalczyk-Quintas, Michaela Seeling, Klaus von der Mark, Pascal Schneider, Holm Schneider

**Affiliations:** ^1^Department of Pediatrics, Friedrich-Alexander University Erlangen-Nürnberg, Erlangen, Germany; ^2^Center for Ectodermal Dysplasias, University Hospital Erlangen, Erlangen, Germany; ^3^Department of Biochemistry, University of Lausanne, Epalinges, Switzerland; ^4^Department of Biology, Friedrich-Alexander University Erlangen-Nürnberg, Erlangen, Germany; ^5^Department of Experimental Medicine I, Friedrich-Alexander University Erlangen-Nürnberg, Erlangen, Germany

**Keywords:** bone, development, ectodysplasin A1, ectodermal dysplasia, fetal therapy, NF-κB

## Abstract

X-linked hypohidrotic ectodermal dysplasia with the cardinal symptoms hypodontia, hypotrichosis and hypohidrosis is caused by a genetic deficiency of ectodysplasin A1 (EDA1). Prenatal EDA1 replacement can rescue the development of skin appendages and teeth. *Tabby* mice, a natural animal model of EDA1 deficiency, additionally feature a striking kink of the tail, the cause of which has remained unclear. We studied the origin of this phenomenon and its response to prenatal therapy. Alterations in the distal spine could be noticed soon after birth, and kinks were present in all *Tabby* mice by the age of 4 months. Although their vertebral bones frequently had a disorganized epiphyseal zone possibly predisposing to fractures, cortical bone density was only reduced in vertebrae of older *Tabby* mice and even increased in their tibiae. Different availability of osteoclasts in the spine, which may affect bone density, was ruled out by osteoclast staining. The absence of hair follicles, a well-known niche of epidermal stem cells, and much lower bromodeoxyuridine uptake in the tail skin of 9-day-old *Tabby* mice rather suggest the kink being due to a skin proliferation defect that prevents the skin from growing as fast as the skeleton, so that caudal vertebrae may be squeezed and bent by a lack of skin. Early postnatal treatment with EDA1 leading to delayed hair follicle formation attenuated the kink, but did not prevent it. *Tabby* mice born after prenatal administration of EDA1, however, showed normal tail skin proliferation, no signs of kinking and, interestingly, a normalized vertebral bone density. Thus, our data prove the causal relationship between EDA1 deficiency and kinky tails and indicate that hair follicles are required for murine tail skin to grow fast enough. Disturbed bone development appears to be partially pre-determined *in utero* and can be counteracted by timely EDA1 replacement, pointing to a role of EDA1 also in osteogenesis.

## Introduction

X-linked hypohidrotic ectodermal dysplasia (XLHED; #MIM 305100), a rare hereditary disease characterized by missing or dysplastic skin appendages and teeth, is caused by deficiency of the signaling protein ectodysplasin A1 (EDA1) during early development (Mikkola and Thesleff, 2003). Numerous mutations in the X-chromosomal gene *EDA* which encodes EDA1 have been reported (Cluzeau et al., 2011; Schneider et al., 2011; Trzeciak and Koczorowski, 2016; Wohlfart et al., 2016), and most of them result in a complete absence of functional EDA1.

Mutations in the genes EDA1 receptor (*EDAR*) or *EDARADD* which underlie genetic deficiencies of the EDAR and its associated death domain-containing adaptor protein, respectively, lead to autosomal recessive or dominant hypohidrotic ectodermal dysplasia. Since all three proteins are part of the EDA1 pathway to nuclear factor (NF)-κB activation, the phenotypic features are similar (Cluzeau et al., 2011). They include hypotrichosis, oligo- or anodontia, and hypo- or anhidrosis. The latter is the most severe disability as it can lead to life-threatening hyperthermia on summer days, during physical activity or febrile illness. This significantly increases the childhood mortality (Blüschke et al., 2010).

Although knowledge about NF-κB signaling has been increasing constantly (Smahi et al., 2002; Zhang et al., 2017), the exact mechanisms by which EDA1 regulates the development of ectodermal structures are still subject to research. After binding of EDA1 to its cognate receptor, the EDA1–EDAR complex recruits the receptor-associated adaptor molecule which activates several tumor necrosis factor receptor-associated factors (TRAFs). TRAF6 builds a complex with TAK1 binding protein 2 (TAB2) and transforming growth factor beta-activated kinase 1 (TAK1) that activates the NF-κB essential modulator (NEMO), leading to phosphorylation and dissociation of the inhibitory complex IκBα from NF-κB. Upon its translocation into the nucleus NF-κB acts as a transcription factor for numerous target genes, e.g., sonic hedgehog (shh) and members of the Wnt protein family (Kowalczyk-Quintas and Schneider, 2014).

The EDA1 pathway is highly conserved among vertebrates, which provides the opportunity of valid research in a mouse model (Pantalacci et al., 2008). The *Tabby* mouse (Ferguson et al., 1997; Srivastava et al., 1997) is a well-established animal model for EDA1 deficiency. Human and murine EDA1 show an amino acid sequence homology of 94% (Monreal et al., 1998). In general, the *Tabby* phenotype reflects the characteristics of human XLHED patients, meaning that hair, teeth, and eccrine glands are affected by the developmental disorder. Different and particularly striking, however, is a characteristic kink in the tail tip of *Tabby* mice which has been reported to result from fractures of vertebral bodies (Hill et al., 2002). Structural abnormalities of both vertebral and tibial bones of *Tabby* mice were described by the same authors (Hill et al., 2002).

In the human embryo, *EDA* is expressed by osteoblasts between gestational weeks 12 and 16 (Montonen et al., 1998). The NF-κB pathway is also known to play an essential role in the differentiation of osteoclasts via RANK-RANKL signaling that involves TRAF6 activation (Clauss et al., 2008). Thus, EDA1 deficiency might have impact on bone development not only in *Tabby* mice.

Various strategies to treat XLHED with an EDA1 replacement protein have been investigated (Gaide and Schneider, 2003; Casal et al., 2007; Hermes et al., 2014). Postnatal protein replacement was partially effective in animal models but not in human patients (Körber et al., 2020), whereas prenatal intra-amniotic administration of a recombinant EDA1 molecule to infants with XLHED resulted in normal sweat gland endowment and sweating ability, development of more teeth, and normalized function of salivary glands (Schneider et al., 2018; Körber et al., 2020) and, thus, showed the potential to permanently resolve the most relevant clinical problems associated with XLHED (Wohlfart et al., 2020). This recombinant protein, composed of the Fc part of human IgG and the receptor-binding domain of EDA1, was named Fc-EDA (Schneider et al., 2018). When swallowed by a fetus in the third trimester of pregnancy, the Fc component facilitates intestinal uptake into the fetal circulation via specific Fc receptors (Schneider et al., 2018).

In this study, we focused again on *Tabby* mice to elucidate the pathogenesis of their kinky tail, the peculiarities of bone development in this animal model, and their response to prenatal therapy with Fc-EDA. We show that intra-amniotic administration of the replacement protein rescues tail skin as well as vertebral bone development and discuss a yet unexplored role of EDA1 in osteogenesis.

## Materials and Methods

### Animal Model

C57BL/6 wild-type mice (Charles River, Sulzfeld, Germany), white-bellied agouti B6CBAa *Aw-J/A-EdaTa/J Tabby* mice (a mouse model for XLHED; Jackson Laboratory, Bar Harbor, ME, United States), and appropriate control mice were housed in individually ventilated cages under standard conditions with a light/dark cycle of 12 h and free access to standard chow and tap water. All experimental procedures on animals were conducted in accordance with German or Swiss regulations and legal requirements (depending on the site of investigation) and had been approved by the local government authorities.

### Therapeutic Interventions

Three litters of newborn *Tabby* mice were treated within 24 h after birth by intraperitoneal injection of Fc-EDA, a recombinant fusion protein consisting of the receptor-binding tumor necrosis factor (TNF) homology domain of EDA1 and the Fc domain of human IgG1 (single dose: 2 mg/kg body weight).

Prenatal administration of Fc-EDA to mouse fetuses was done as described before (Hermes et al., 2014; Schneider et al., 2018). Briefly, pregnant time-mated *Tabby* mice were anesthetized with isoflurane (2%) and analgized during and after surgery on gestational day 15 (E15) with metamizole (subcutaneous injection of 5 mg). A laparotomy was performed, followed by complete exposure of both uterine horns. Fc-EDA was injected into the amniotic sacs of individual fetuses at doses of 100 μg/g fetal body weight (16 μl of Fc-EDA at 2.2 mg/ml, assuming a fetal weight of approximately 0.35 g) using a glass syringe with 33 gauge needle (Hamilton Robotics, Bonaduz, Switzerland). After repositioning of the uterine horns, the abdominal cavity was closed by suturing peritoneum and skin separately (Schuepbach-Mallepell et al., 2020). Treated animals were sacrificed at the age of 2 months or above by cervical dislocation under isoflurane anesthesia. Their tails were collected and investigated.

### Micro-Computed Tomography

After removing all muscle tissue, tibial, and vertebral bones were fixed in paraformaldehyde solution (4%) for 24 h. Images of these bones at 50 μm-resolution were then acquired on an Inveon Micro-PET/CT-Scanner (Siemens, Erlangen, Germany) at the Preclinical Imaging Platform Erlangen. Cross-sections were evaluated with respect to the volume of the medullary cavity and cortical bone density, measured in Hounsfield units, using the DICOM viewer Osirix (Würzburg, Germany). Macroscopic images shown in this paper are based on virtual three-dimensional reconstructions.

### Whole-Mount Skeletal Staining of Embryos and Newborn Pups

Embryos of untreated *Tabby* and wild-type mice at gestational days 16.5 (E16.5) and 18.5 (E18.5) and pups at postnatal days 0 (P0), 4 (P4), 7 (P7), and 21 (P21) were investigated for tail axis deviations and sacrificed by decapitation at the time points indicated. Skin, internal organs and adipose tissue were detached to facilitate permeabilization of the target tissue. The embryos/pups were fixed in 70% ethanol at 4°C overnight, transferred to 95% ethanol for 1 h, followed by incubation in acetone at room temperature overnight and subsequent staining with Alcian blue solution for 1–4 h (embryos) or overnight (pups), respectively. Embryos were cleared in KOH (1%) and glycerol-KOH (1%) solution (equal parts) for 12 h each and finally transferred to 100% glycerol for long-term storage. In newborn pups, unspecific Alcian blue staining was removed by washing initially with 70% ethanol (two changes), followed by incubation in 95% ethanol overnight prior to the KOH clearing described above. Stained whole-mount skeletal tissue sections were investigated using a stereomicroscope MZ 10F (Leica Microsystems, Wetzlar, Germany) with Zeiss Axiocam Typ MRc and Axiovision 4.7 software (Carl Zeiss Microscopy GmbH, Göttingen, Germany).

### Histological Assessments and Trabecular Bone Histomorphometry

Vertebral and tibial bones of older pups or adult mice were decalcified for 7 days in 14% (wt/vol) EDTA (pH adjusted to 7.2 by addition of ammonium hydroxide) and then embedded in paraffin. Vertebral bones (tails) were cut into 5-μm sections, tibiae into 8-μm sections, respectively. For morphological assessment, tissue sections were stained with hematoxylin and eosin. Alcian blue staining was used to visualize the cartilaginous components of the epiphyseal growth plate. In order to evaluate trabecular bone parameters and the amount of osteoclasts, tissue sections were stained for tartrate-resistant acid phosphatase using the leukocyte acid phosphatase (TRAP) kit 387A (Sigma-Aldrich, St. Louis, MO, United States) according to the manufacturer’s instructions. All parameters were quantified by digital image analysis (OsteoMeasure; OsteoMetrics, Decatur, GA, United States).

### Bromodeoxyuridine Incorporation Assays

Wild-type and *Tabby* mouse pups at day P9 were treated by intraperitoneal bromodeoxyuridine (BrdU) injection (40 mg/kg body weight) 2 h before sacrificing them by decapitation (BrdU stock of 5 mg/ml; Sigma-Aldrich, Taufkirchen, Germany). The tails were fixed in 4% paraformaldehyde solution and embedded in paraffin. Sections were deparaffinized, rehydrated, subjected to antigen retrieval in citrate buffer and digestion with 0.025% protease (Type V; Sigma-Aldrich) and then subjected to immunofluorescence staining using a rat monoclonal antibody against BrdU (Abcam, Cambridge, United Kingdom). A donkey anti-rat IgG antibody conjugated to Alexa Fluor 568 (Abcam) was applied for BrdU detection. Cell nuclei were counter-stained with 4′,6-diamidino-2-phenylindole dihydrochloride (DAPI; Sigma-Aldrich). The resulting images were investigated and photographed under a fluorescence microscope (Axio Observer; Zeiss, Jena, Germany). BrdU-positive cells were counted in epidermis and dermis except for the hair bulb region. Data were obtained from six independent sections of each mouse.

### Statistical Analyses

Datasets were analyzed with unpaired *t*-tests using the GraphPad Prism software 7 (GraphPad Software Inc., La Jolla, CA, United States).

## Results

### Embryonic and Postnatal Development of the Caudal Spine in *Tabby* Mice

As the kinky tail tip, combined with the absence of tail and guard hair, is a characteristic feature of adult *Tabby* mice (Kowalczyk-Quintas et al., 2014), we first determined the age range in which the kink appears. Macroscopic assessment (Figures 1A,B, upper panels) and whole-mount Alcian blue staining (Figures 1A,B, lower panels) of the tails did not indicate abnormal development of vertebrae in the caudal spine of *Tabby* mice until birth. Tail tips of *Tabby* mouse embryos at days E16.5 (not shown) and E18.5 (Figure 1B), were indistinguishable from those of wild-type animals (Figure 1A). Deviations from the otherwise linear tail axis were observed in 13 of 29 *Tabby* mice (45%) at day P4. Their frequency and extent increased during the next 3 weeks (Figure 1B). Kinks were present in the tails of all *Tabby* mice by the age of 4 months (data not shown). The vertebral bodies at the kink position seemed to be shortened compared with the surrounding ones, yet until day P21 no obvious fracture was noticed.

**FIGURE 1:**
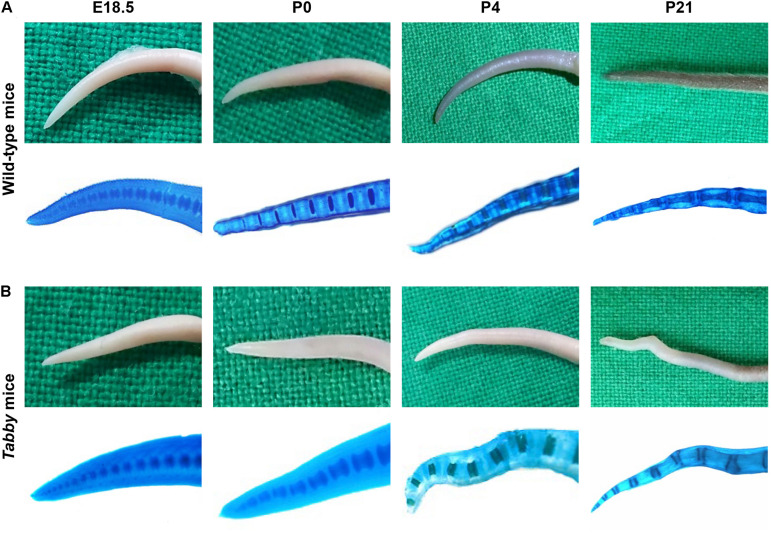
*Tabby* mice develop vertebral dislocations soon after birth. Macroscopic comparison of representative tail tips from **(A)** wild-type and **(B)** untreated *Tabby* mice at embryonic day E18.5 and postnatal days P0, P4, and P21. Lower panels in panels **(A,B)** depict whole-mount staining of cartilaginous parts of the same tails with alcian blue. In nearly half of the *Tabby* mice, first alterations of the distal spine could be detected already on day P4; kinks were evident in 21 of 23 animals investigated at day P21 and in all *Tabby* mice by day P120.

### Cortical Bone Densities of *Tabby* Mice and Osteoclastogenesis

Tails of adult *Tabby* and control mice (2–12 months old) were investigated by micro-computed tomography (CT). Three-dimensional reconstructions of the caudal spine showed fractured vertebral bodies in most of the *Tabby* mice (Figure 2A), sometimes even two or more fractures in one vertebral body. Usually the third to sixth vertebrae, counted from the tail tip, were affected. These fractures which most likely occurred at some point between day P21 and the time of tomography could have been due to structural abnormalities of the bone. The tibiae of wild-type and *Tabby* mice (Figure 2B) did not show macroscopic differences. Histological examination of vertebral and tibial bone sections of the same animals revealed an apparent loss of the longitudinal orientation of vertebral bodies (Figure 2C). Their epiphyseal growth plate was still recognizable, but smaller and less well organized with respect to the chondrocytes’ zonal arrangement (Figures 2C,E) than in wild-type animals or in the tibia of *Tabby* mice (Figures 2D,F) and contained numerous cartilage islands.

**FIGURE 2:**
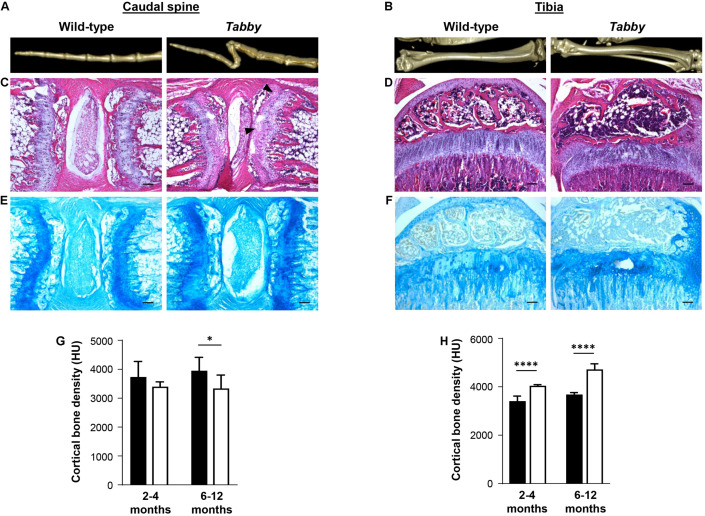
Impact of ectodysplasin A1 (EDA1) deficiency on osteogenesis and bone density. **(A,B)** Representative three-dimensional reconstructions of **(A)** the distal tail portion and **(B)** tibiae of wild-type and *Tabby* mice. **(C–F)** Vertebral and tibial bone sections stained with hematoxylin-eosin **(C,D)** or alcian blue **(E,F)**: The epiphyseal region appears less well organized and contains more cartilage islands (arrowed) in the tails of *Tabby* mice than in wild-type mice. **(G)** Although computed tomography scanning (micro-CT) revealed a tendency toward reduced cortical bone density already in tails of *Tabby* mice at an age of 2–4 months (mean of 7–10 distal vertebrae where the kink is formed; indicated in Hounsfield units), this difference became significant only in *Tabby* mice older than 6 months (*n* = 6; white bars) compared with wild-type animals (*n* = 6; black bars). **(H)** Tibial bones, in contrast, showed a higher cortical density in *Tabby* mice of both age groups (*n* = 6 and *n* = 7, respectively; white bars) than in wild-type animals (black bars) as determined by micro-CT measurements. Scale bar in panels **(C–F)**: 100 μm. Data are shown as mean ± SD; ^∗^*p* < 0.05; ^****^p < 0.0001.

Micro-CT measurements of 7–10 vertebral bodies indicated a significantly higher volume of the medullary cavity in *Tabby* mice compared with wild-type animals (0.405 ± 0.028 vs. 0.290 ± 0.0274 mm^3^, *p* = 0.004), while cortical bone density appeared to be comparatively low in *Tabby* mice aged 2–4 months and was significantly reduced in older *Tabby* mice (Figure 2G). Tibial bones, in contrast, were found to have a higher cortical density in *Tabby* than in wild-type animals (Figure 2H), irrespective of their age. Thus, EDA1 deficiency had an impact on bone density.

Microscopic assessment of TRAP-stained sections of wild-type and *Tabby* mouse vertebrae indicated a similar amount of osteoclasts in the metaphyseal trabecular bone (Figure 3A). We focused on osteoclasts, because the EDA1 pathway is known to interact with the RANKL/RANK signaling pathway (Figure 3B) that plays an important role in osteoclastogenesis (Wada et al., 2006). Trabecular bone histomorphometry using digital image analysis software was performed: The number of osteoclasts normalized to the trabecular bone perimeter in the metaphyseal part of vertebrae was comparable between *Tabby* and wild-type mice (3.41 ± 1.28 vs. 3.19 ± 0.82, respectively; five animals per group investigated). Although trabecular separation appeared to be larger in the vertebral bones of some *Tabby* mice, in agreement with the higher volume of the medullary cavity revealed by micro-CT measurements, neither trabecular number nor separation differed significantly between *Tabby* and wild-type mice.

**FIGURE 3:**
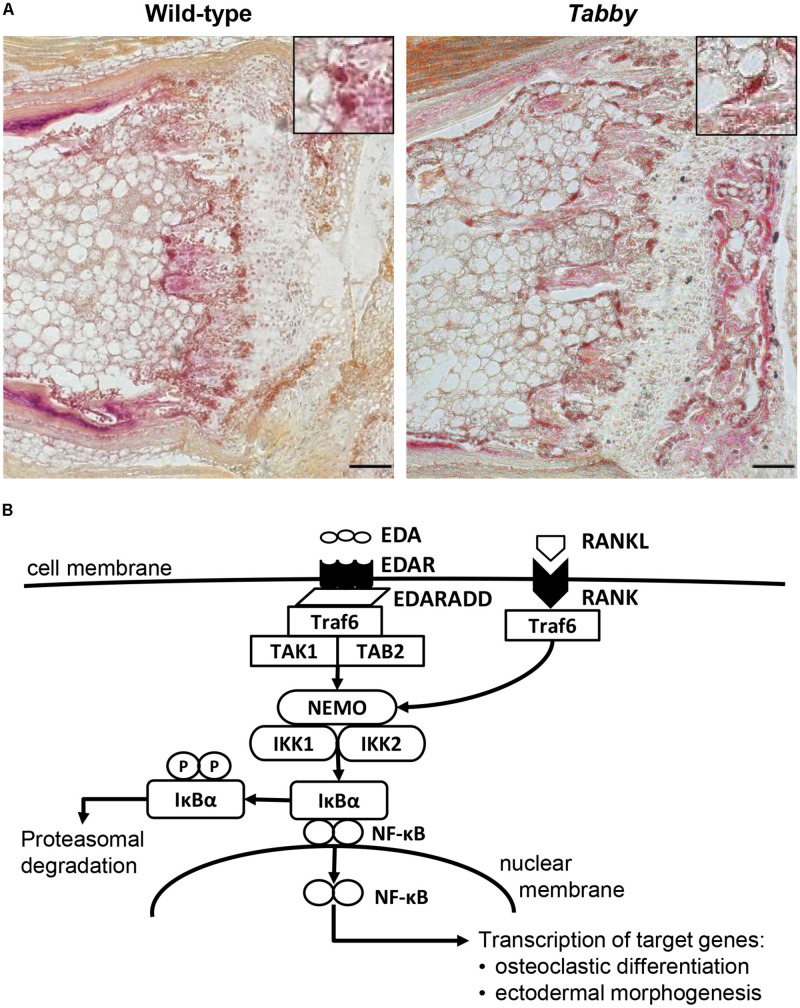
Possible role of EDA1 in the differentiation of osteoclasts. **(A)** Representative tartrate-resistant acid phosphatase (TRAP)-stained tail sections of wild-type and *Tabby* mice indicating similar number and distribution of osteoclasts in the metaphyseal trabecular part of vertebral bones. The boxes in the upper right corner show magnified osteoclasts (dark red). **(B)** Overview of the molecular link between the EDA1 pathway and the RANK–TRAF6–NFκB pathway and its possible involvement in osteoclastic differentiation. Scale bar: 100 μm.

As the absence of significant differences in vertebral cortical bone density between wild-type and *Tabby* mice at the time when the kink appears did not support an important role of bone density in the formation of the kink, an alternative hypothesis needed to be investigated: the possibility that the tail skin does not grow fast enough and the pressure on the vertebral bodies at the tip of the tail bends them, until this is “fixed” by some kind of sclerosis. Kinks would then form close to the tail tip, because the pressure there will be higher and the vertebral bones smaller and easier to deform.

### Insufficient Growth of the Tail Skin in *Tabby* Mice

In order to assess the skin growth at the tails of newborn animals, we performed a BrdU incorporation experiment at day P9 and compared the numbers of BrdU-positive cells in tail skin sections of wild-type and *Tabby* mice (Figure 4). Our data clearly show a diminished keratinocyte proliferation in the epidermis of *Tabby* mice and an absence of hair follicles (Figure 4A), the main source of epidermal stem cells. The number of BrdU-positive cells in the epidermis of wild-type mice was more than two-fold higher (*p* < 0.0001; Figure 4B). A similar difference was observed with respect to BrdU-positive cells in the upper dermis of *Tabby* and wild-type mice except for the hair bulb region (Figure 4C).

**FIGURE 4:**
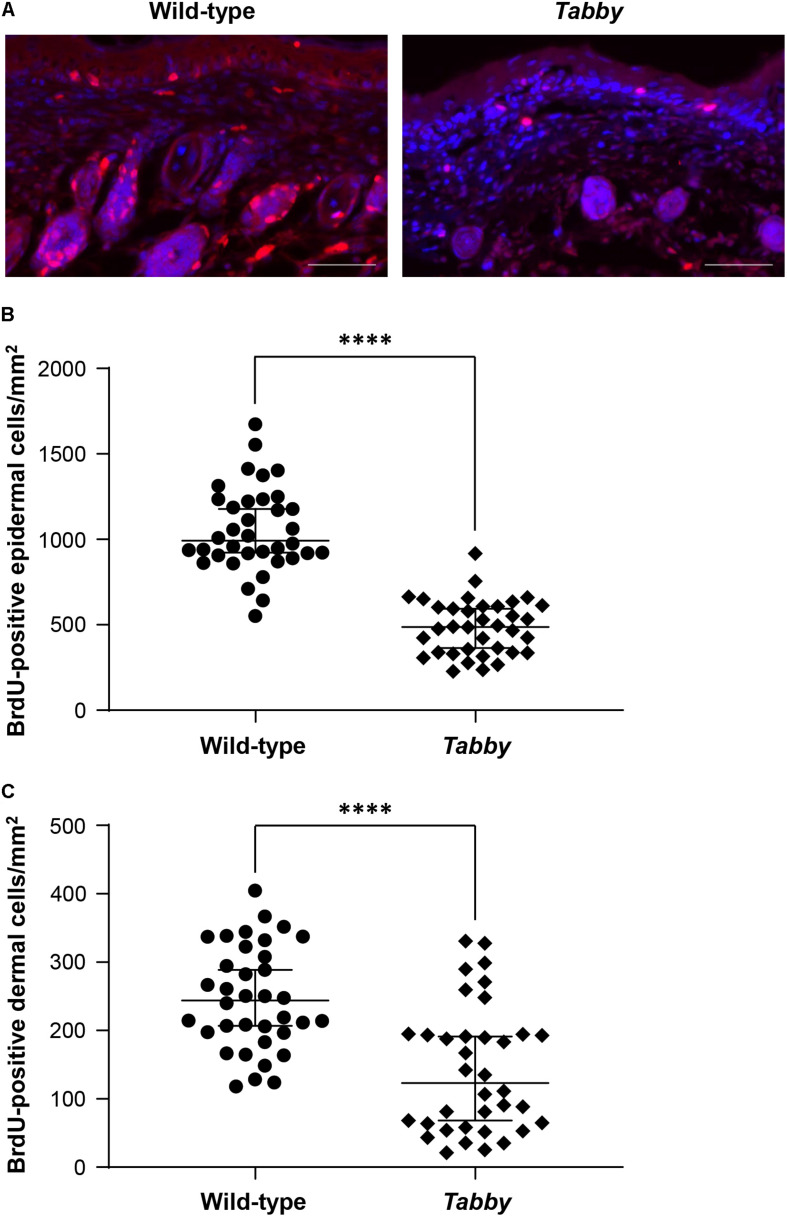
Diminished proliferation of tail skin in *Tabby* mice. **(A)** Immunofluorescence staining of tail tip cross-sections from wild-type (*n* = 6; *left panel*) and *Tabby* mice (*n* = 6; *right panel*) after intraperitoneal injection of bromodeoxyuridine (BrdU) at day P9 to label replicating cells. The amount of BrdU-positive cells in the skin (red; nuclei counter-stained blue with DAPI) was assessed. **(B,C)** Quantitation of replicating cells visualized by BrdU incorporation. BrdU-positive nuclei were counted in the epidermis and the upper dermis (depth ≤ 0.15 mm) except for the hair bulb region. Data from six independent skin sections of each *Tabby* and wild-type mouse are shown. Scale bar: 50 μm. ^****^p < 0.0001.

When *Tabby* mice were treated by intraperitoneal injection of Fc-EDA (2 mg/kg body weight) within 24 h after birth, kink formation–quantified by measuring cumulative angles at the tail tip (Figure 5A)–was diminished but not completely prevented (Figure 5B), while delayed normal tail hair development occurred. As several days are probably required for the development of hair follicles after a triggering signal and as tail kinks had been observed as early as on day P4 in untreated *Tabby* mice, these data fit well with the hypothesis that such kinks form because a skin devoid of hair follicles does not grow fast enough to accommodate the incessantly growing caudal vertebral bodies in the first weeks of life.

**FIGURE 5:**
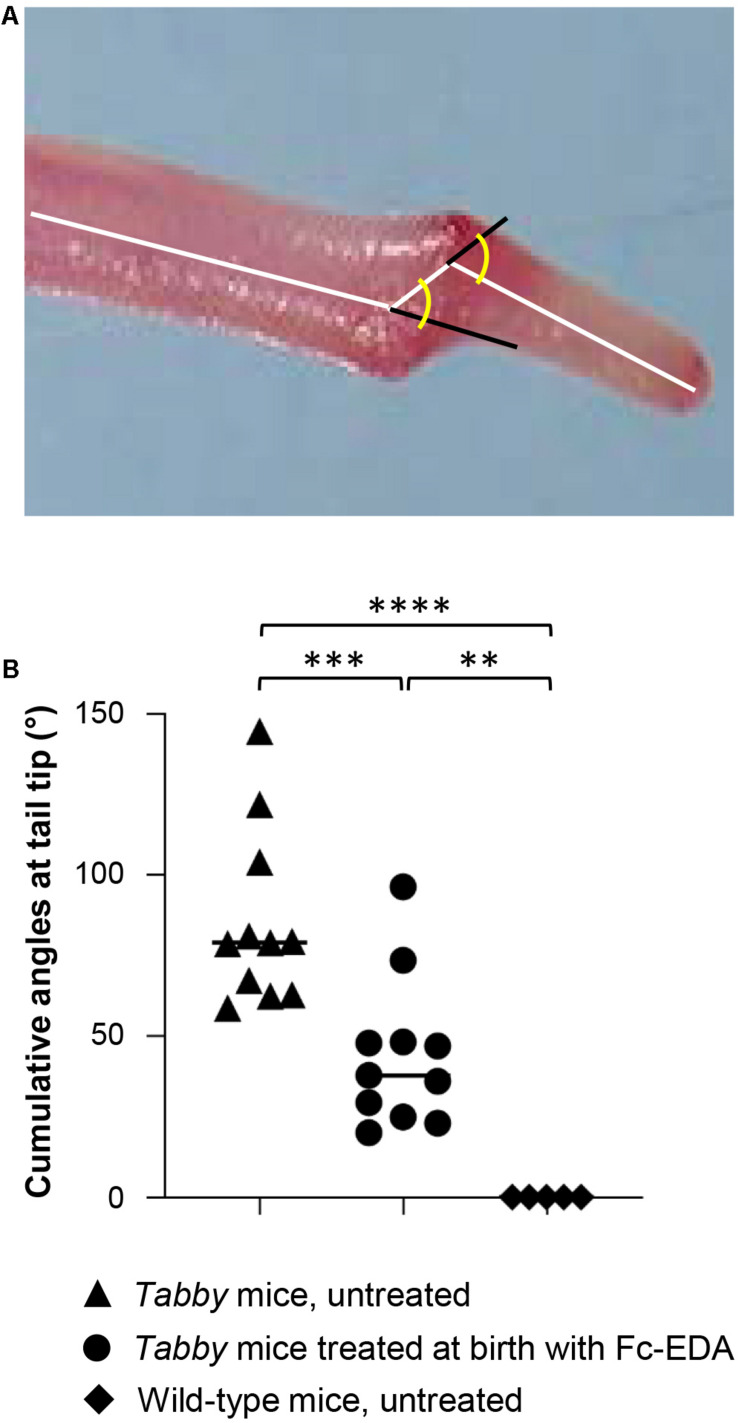
Attenuation of the tail kink in *Tabby* mice upon postnatal treatment with EDA1. **(A)** *Tabby* mice were treated at birth with EDA1 (2 mg/kg body weight, intraperitoneal injection) and sacrificed at weaning. Pictures of the tails were taken for measurements. The amplitude of kink was quantified by the cumulative angles at the tail tip using ImageJ. **(B)** Cumulative angles (°) of the tail tips of untreated *Tabby* mice (*n* = 11; black triangles), *Tabby* mice treated at birth with EDA1 (*n* = 11; black circles) and untreated wild-type mice (*n* = 5; black squares). Data are shown as mean ± SD; ^∗∗^*p* = 0.001–0.01; ^∗∗∗^*p* = 0.0001–0.001; ^****^p < 0.0001.

Tail hair development can, however, be rescued even at an earlier stage by administration of Fc-EDA *in utero* (Hermes et al., 2014). Such prenatal treatment of *Tabby* mice on day E15 indeed resulted in a completely corrected phenotype (Figures 6A,B) with a kink-free tail resembling that of wild-type animals. This is in full agreement with the findings described above and confirms our hypothesis that hair follicles are required for a sufficient growth of murine tail skin. Furthermore, and perhaps most interestingly, prenatal EDA1 replacement also led to a normal cortical bone density in the caudal vertebral bodies of *Tabby* mice aged 6–12 months (Figure 6C).

**FIGURE 6:**
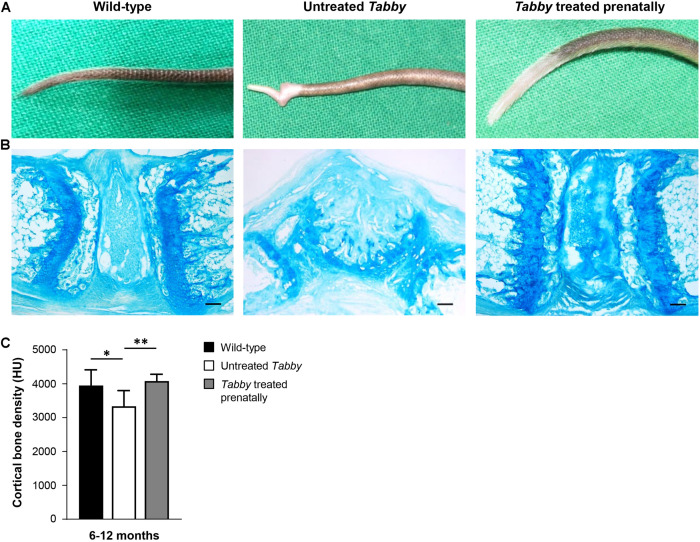
EDA1 replacement *in utero* corrects vertebral bone development in *Tabby* mice. **(A)** Representative tails of wild-type, untreated, and prenatally treated *Tabby* mice. **(B)** Tail tip sections from the region of the kink stained with alcian blue (scale bar: 100 μm). **(C)** Vertebral bone density (Hounsfield units) of wild-type mice (*n* = 6; black bar), untreated *Tabby* mice (*n* = 6; white bar) and *Tabby* mice treated *in utero* (*n* = 7; gray bar). All animals were 6–12 months old. Data are shown as mean ± SD; ^∗^*p* < 0.05; ^∗∗^*p* = 0.001–0.01.

## Discussion

The results of our study confirm previously reported observations by another group (Hill et al., 2002) that EDA1 deficiency entails bone defects in mice. In particular, adult *Tabby* mice display fractures of vertebral bodies in the caudal spine. They seem to develop osteoporotic vertebral bodies – characterized by reduced cortical bone density – earlier than wild-type mice. This, however, may be caused by a reduced mobility of older *Tabby* mice (unpublished own observation) rather than by defective EDA1 signaling. In any case, osteoporosis does not explain the kinky tails regularly observed during the first weeks or months of life.

On the other side, the results of our prenatal rescue experiment indicate that although reduced bone density is only seen in aged EDA1-deficient mice, it may already be pre-determined *in utero* and, if not, is at least counteracted by something that can be induced by timely EDA1 replacement. NF-κB, the transcription factor activated by the EDA1 signaling cascade, is a major regulator of skeletal development and osteoclastic differentiation, requiring activation of the RANKL–RANK–TRAF6 complex (Franzoso et al., 1997; Smahi et al., 2002; Asagiri and Takayanagi, 2007). Impairment of this pathway in *Tabby* mice might interfere with the EDA1–TRAF6–RANK molecular interaction and, thus, with osteoclastic differentiation (Clauss et al., 2008). The EDAR was found to be expressed in osteoclasts (unpublished own data). Osteoclasts in *Tabby* and control mice did not differ in number, but their activity might be different. NF-κB is known to play a critical role also in the maturation of osteoclasts (Clauss et al., 2008). As TNF can synergize with minute amounts of RANKL to promote osteoclastogenesis (Lam et al., 2000), EDA1 might also be able to synergize with RANKL, which could be a subject of further studies. In addition, future research may use relevant mesenchymal precursor cells, such as bone marrow stromal cells, from *Tabby* mice to investigate their osteogenic differentiation *in vitro* (Gelse et al., 2003), aiming at a careful characterization and evaluation of the activity of osteoblasts and osteoclasts.

Kinky tails have been observed in various mouse models (Wright, 1947; Schrick et al., 1995; Farkas and Chapman, 2009) and may be related to a second, independent mutation in genes involved in the development of the spine. The latter is highly unlikely in the case of *Tabby* mice, because the effect of an independent mutation would probably not be correctable by treatment with Fc-EDA and should not be sensitive to the time of its administration.

Previous work of our group demonstrating the generation of *Tabby*-like mice by treatment of wild-type animals with anti-EDA1 antibodies (Kowalczyk-Quintas et al., 2014) suggested restricted skin growth in *Tabby* mice to be at least part of the explanation for the kinky tail. Also, in a related mouse model, hair follicles were found to be required for optimal growth during lateral skin expansion and their absence was shown to lead to shortening and kinking of the caudal spine (Heath et al., 2009). The authors concluded that skin growth was unable to keep pace with the rapidly elongating axial skeleton of the tail. We focused on this hypothesis, because wild-type mice treated perinatally with the anti-EDA1 antibody EctoD2 got a kinky tail (Kowalczyk-Quintas et al., 2014), albeit late appearance of tail hair indicated that the intermittent treatment was not inhibiting EDA1 all the time, so that hair follicles could develop with some delay.

Early postnatal treatment of *Tabby* mice with Fc-EDA attenuated but did not completely prevent the kink. This can also be explained by the delayed rescue of hair follicles in the tail skin. Only prenatal administration of Fc-EDA may ensure full correction of the skin’s capacity to grow fast enough. Induction of tail hair at a later stage does not solve the problem at the initial step of kink formation, but thereafter allows the skin to grow faster, leading to a smaller amplitude of the kink.

Thus, our prenatal rescue experiment demonstrates clearly the causal relationship between EDA1 deficiency and kinky tails. To what extent the partially disturbed bone development evident in aged *Tabby* mice is pre-determined in the embryo, pointing to a role of EDA1 also in osteogenesis, needs to be investigated in further studies. As the bone phenotype in *Tabby* mice appears to be due to the lack of EDA1, not EDA2, it is most likely EDAR-dependent. The implication of this receptor could be assessed using conditional EDAR-knockout mice (Vial et al., 2019) crossed with bone-specific Cre deleter mice (Dallas et al., 2018) to better understand a direct or indirect contribution of EDAR signaling to bone development and which cell types are involved.

## Data Availability Statement

The raw data supporting the conclusions of this article will be made available by the authors, without undue reservation.

## Ethics Statement

Animal experiments performed in this study were approved either by Regierungspräsidium Karlsruhe and Regierung von Unterfranken (authorization 55.2-2532-2-222 to HS) or by the local institutional Animal Care and Use Committee and by the Office Vétérinaire Cantonal du Canton de Vaud (authorization 1370.7 to PS).

## Author Contributions

PS and HS conceived the experiments and supervised the work. C-SK, MW, SS-M, JP, CK-Q, and MS performed laboratory investigations. KM curated data. C-SK and HS wrote the first draft of the manuscript. All authors critically reviewed the manuscript and approved its final version.

## Conflict of Interest

PS, CK-Q, and HS are inventors on patents relevant to this publication. The remaining authors declare that the research was conducted in the absence of any commercial or financial relationships that could be construed as a potential conflict of interest.

## Publisher’s Note

All claims expressed in this article are solely those of the authors and do not necessarily represent those of their affiliated organizations, or those of the publisher, the editors and the reviewers. Any product that may be evaluated in this article, or claim that may be made by its manufacturer, is not guaranteed or endorsed by the publisher.
